# Developing a management and finance training for future public health leaders

**DOI:** 10.3389/fpubh.2023.1125155

**Published:** 2023-05-11

**Authors:** Kristy T. Hayes, Harry J. Heiman, Peggy A. Honoré

**Affiliations:** ^1^School of Public Health, Georgia State University, Atlanta, GA, United States; ^2^School of Public Health and School of Medicine, Louisiana State University Health Sciences Center New Orleans, New Orleans, LA, United States

**Keywords:** public health, management, finance, Doctor of Public Health, workforce capacity building, management and accounting

## Abstract

Public health leaders are increasingly being asked to address adaptive challenges in the context of finite and often limited resources. Budgets and their associated resources create the financial framework within which public health agencies and organizations must operate. Yet, many public health professionals expected to undertake roles requiring this foundational knowledge and skills are not trained in the fundamentals of public finance and are ill-equipped for managing and monitoring funds. Graduate courses in schools of public health most often are focused on health care management and finance or private sector finance. To meet the needs of future public health leaders, it is critical that academic content builds capacity in management and finance focused on public health practice. This paper describes the development of a Doctor of Public Health program management and finance course designed to prepare future public health leaders. The course aims to build the knowledge and skills of doctoral-level students to recognize the inherent challenges of public health finance and the importance of cultivating and managing resources to improve public health practice and achieve strategic public health goals.

## Introduction

Public health management and finance training is critical to the preparation of public health professionals and leaders. Public health leaders are increasingly being asked to address adaptive challenges in the context of finite and often limited resources. Budgets and their associated resources create the financial framework within which public health agencies and organizations must operate. Yet, many public health professionals are expected to undertake roles requiring this foundational knowledge and skills are ill-equipped for managing and monitoring funds because they were not trained in the fundamentals of public finance (1). A 2008 analysis found that there were no U.S. graduate public health programs that required a finance course for public health non-management concentration students (1). In addition, most of those who had taken a management and finance course were trained about health care management and finance or private sector finance, neither of which prepares students for public health practice (2). Based on a 2009 national survey, Honoré and Costich detailed a list of public health finance competencies which covered three domains including financial management, analysis, and assessment; organization and program planning and policy development; and administrative skills (2). The gap between what is taught in graduate public health training programs and the requirements for effectively and efficiently allocating and managing resources in public health practice is especially magnified for those assuming management and leadership roles. Strengthening the skills and knowledge of graduate students based on these competencies is essential preparation for public health practice.

The Doctor of Public Health (DrPH) degree was developed to provide doctoral-level training to prepare public health leaders for applied public health practice. In contrast to the Doctor of Philosophy (PhD) degree in public health, which is targeted to preparing students for careers in research, DrPH training is designed to prepare “a transformative leader with expertise in evidence-based public health practice and research who is able to convene diverse partners, communicate to affect change across a range of sectors and settings, synthesize findings, and generate practice-based evidence” (3). The development of DrPH programs grew from the acknowledgment that many leadership positions in public health are held by medical doctors and others without the necessary training or skills to lead agencies or organizations focused on population health (3). A 2014 report from the Association of Schools and Programs of Public Health (ASPPH) includes fiscal responsibility as critical content for DrPH programs to stay relevant in the 21^st^ Century (4). Competencies closely aligned with public health management and finance comprise three of twenty foundational DrPH competencies required for Council on Education for Public Health (CEPH) accreditation, including the ability to propose human, fiscal, and other resources to achieve a strategic goal; cultivate new resources and revenue streams to achieve a strategic goal; and propose interprofessional and/or intersectoral team approaches to improving public health. Fiscal stewardship, including sustainable and flexible funding, is also a core element for Public Health 3.0, a forward-looking model for public health leadership and practice that recognizes the critical importance of cross-sector collaboration to address upstream social determinants of health (5). For public health to evolve in a way that both learns from the experiences of the COVID-19 pandemic and meaningfully prioritizes advancing health equity, practicing professionals will need to learn innovative strategies and models of funding to stabilize revenue streams and address social determinants of health (5). This will require training diverse and skilled leadership prepared to blend and braid funds to support multi-sectoral collaboration (6), advocate for sustainable public health funding, and match resources to need, targeting those communities experiencing the greatest inequities. This paper describes the development of a DrPH Public Health Finance and Management course. We provide a description of the development of the curriculum, lessons learned after the first year of implementation, and a discussion on the practical applications for the field of public health.

## Pedagogical framework

We took a three-pronged approach to developing a course on public health management and finance targeted to DrPH students. This included reviewing the literature on course design and adult learning, reviewing the current literature on public health management and finance, and finally assessing other schools' doctoral-level curricula on management and finance.

We reviewed the literature on course design, learning objectives, and approaches to learning and teaching. Because of the diverse backgrounds of DrPH students, including educational backgrounds, work experience, and levels of experience in finance, the literature on higher education allowed us to bring together theory and practice to strengthen academic content for adult learners. Two theoretical constructs from Light and Cox's Learning Gap Framework and Bloom's taxonomy of educational objectives were used to inform pedagogical approaches for adult learners. The Learning Gap Framework acknowledges approaches to learning and strategies needed for adults who learn different things in different ways (7). Given the overarching goal of preparing public health leaders for applied public health practice, the class structure, activities, and assignments created the space for creativity and innovation in lecturing. Developing the interpersonal construct through peer learning embeds the Learning Gap Framework to close the gap of learning content with the intent to achieve ongoing change (8). Students are placed into groups of 3 to 5 students based on a self-assessment of their own experience and background in management and finance. Groups are made to encourage contributing and learning from others based on student's diverse exposure in management and finance and group size guarantees that all students will have a good opportunity to contribute to the lesson or activity. The learning objectives of each lesson also aim to strengthen skill sets through Bloom's learning spectrum of knowledge, comprehension, application, analysis, synthesis, and evaluation, with an emphasis on higher level application and synthesis (9). These taxonomies supported the development of the curriculum and delivery approaches to help course instructors conduct real-time assessments of students' knowledge and skill levels.

To inform course content, we conducted an online search of the literature, web sites, and presentations. Using search terms, “public health finance; public health management and finance; health finance; public policy management; nonprofit management; public health budget; government finance; government budget,” we reviewed results from PubMed, Google Scholar, and Google. From this online scan, we compiled publications, presentations, roundtable discussions, and a training bootcamp specifically on public health management and finance. Many of the materials we found were products from a 2009 effort supported by the Robert Wood Johnson Foundation to optimize public health management and finance training and education (10). This work generated a significant number of resources that greatly informed this curriculum.

We also examined graduate-level management and finance courses and their descriptions in schools of public policy and public health in the United States. Schools of public policy were included because of their academic attention on public finance. We reviewed the course catalogs and syllabi for the top 20 schools of public health with DrPH programs and top 10 public policy schools in the U.S. as rated by *U.S. News and World Report* in 2020 (11). A search for management and finance courses in the schools' course catalogs was performed using the same search terms as the online search above. Of the 30 schools, four schools did not have course catalogs accessible online and an additional four schools did not yield any courses using our search terms. From the remaining 22 schools we identified 37 applicable courses. Nine of the 37 courses reviewed were relevant to public management and finance but were not specific to public health and 13 courses contained the search terms but either a course description was not available, or the course was primarily focused on health care management and finance. The remaining 15 courses had a course description or learning objective related to public management and finance, of these 24% originating from public policy schools and 16% from schools of public health. These courses offered the fundamentals of accounting principles, an introduction to financial management and managerial accounting, examined public finance policy, and health economics theoretical concepts. While this content is an important foundation for DrPH students, it almost exclusively focused on the application in health care settings, not specifically in public health.

While acknowledging the value of accounting skills and the intersections of health care and public health, we developed a curriculum targeted to preparing practicing public health professionals and leaders across a range of settings and roles. As reflected in the foundational competencies, DrPH training should prepare public health professionals to engage in leadership-level budget discussions and provide both critical input and analysis. The course learning objectives are based on the CEPH DrPH foundational competencies and each lesson is associated with the overall course objective, including:

- #12: Propose human, fiscal and other resources to achieve a strategic goal.
- #13: Cultivate new resources and revenue streams to achieve a strategic goal.
- #17: Propose interprofessional and/or intersectoral team approaches to improving public health.

The purpose of the course is to prepare public health practitioners for real-world application of management and finance in public health agencies and organizations. This course provides an overview of basic accounting concepts to build skills in preparing, analyzing, and implementing budgets and applies management decision-making strategies to inform public health programs and policies. The intended learning outcomes are to share an array of perspectives of finance and management at the local, state, federal, and global levels, build the knowledge base on accounting principles, and critically apply team building and management skills.

The lessons and learning objectives from the draft curriculum were shared through an online survey with 16 selected experts in the field of public health management and finance to validate course content. Experts were identified by reviewing authorship of peer-reviewed publications and advocates of public health management and finance. Lastly, an online survey was completed by 20 out of 30 currently enrolled Georgia State University DrPH students to assess their backgrounds and needs relative to the content areas. Feedback was provided by both experts and students and incorporated into the curriculum.

With consensus on the lessons, a textbook or book chapter(s) on public health management and finance was not identified. We conducted a scan online using key terms from the learning objectives to identify relevant peer reviewed publications, contemporary news articles, webinars, and videos. A variety of modes and relevant or contemporary resources were selected to uphold adult learning.

## Learning environment

This course was initially implemented in a remote synchronous format during the 2021–2022 academic year to 23 DrPH students. An adjunct faculty member who was a recent DrPH program graduate with an educational background in accounting and economics and over 10 years of public health experience taught this course in Georgia State University School of Public Health's DrPH program. The learning objectives across 13 lessons aimed to provide a foundation for public health management and finance with real-world application. It also intentionally integrated a health equity lens to both concepts and examples related to both allocation of resources and targeting of funds to socially, economically, and/or environmentally disadvantaged populations experiencing health inequities. The course also incorporated principles of blending and braiding funds to support cross-sector collaboration and address upstream social determinants of health. Table 1 is the list of sessions, learning objectives, and corresponding CEPH competencies. Lessons 1–4 build on foundational skills, recognizing most DrPH students do not have backgrounds in business or accounting, to learn basic principles and terminology. Lessons 5–13 were informed by the literature on public health management and finance. The full curriculum, found in Appendix A, provides the description, learning objectives, and readings.

**Table 1:** Public health finance and management learning objectives and CEPH competency.

**Lesson**	**Learning objectives**	**CEPH competency**
Introduction	•Summarize fundamental theories, concepts, and definitions of public health finance. •Apply strategies for public health financing in Public Health 3.0. •Self-evaluate personal experiences and expectations to improve knowledge and skills in finance and management.	12
Planning and budgeting	•Discuss Congressional appropriation process in theory and practice. •Analyze differences in U.S. state public health funding levels. •Critique funding mechanisms and types of revenue sources for federal, state, and local public health.	12
Operating budgets	•Evaluate the overall planning process and develop an operating budget. •Design a budget using different types of costs and budget assumptions. •Analyze a planning and operating budget.	12
Financial statements	•Calculate break-even analysis and discounted cash flow analysis. •Evaluate financial statements and key players in financial statement regulation. •Design a planning budget for a public health program.	12
Taxation	•Evaluate taxation as a revenue source to support public health goals. •Compare and contrast states with different tax structures and uses of revenue. •Design an operational budget for a public health program	13
Revenue generation	•Explain social impact bonds and develop a real-world example. •Critically examine sustainability and utility of new economic models. •Design a budget that blends and braid funds to address upstream social determinant of health.	13
Spending and return on investments	•Discuss evidence-based resource allocation processes. •Examine real world resource allocation processes. •Apply equity in a resource allocation decision-making process.	17
Fiscal stewardship and transparency	•Examine public health expenditure data sources. •Assess the relationship between resource allocation and health equity goals. •Critique funding policy using the Tobacco Master Settlement Agreement as an example.	13
Partnerships	•Discuss fiscal stewardship, accountability, and transparency of public health funds. •Critique public health programming and closing the finance gap. •Compare and contrast state-level expenditures in social services and social determinants of health.	13
Decision-making strategies	•Analyze Medicaid's role in partnering with public health. •Evaluate the intersection between public health and safety net programs to improve social determinants of health. •Assess public health agencies' ability to blend funds to support people living with disabilities.	17
Ethics	•Examine the influence of philanthropic and private industry funds in public health. •Evaluate the intersection of pharmaceutical research and public health research and practice. •Critique the decision-making process in accepting funds to address a public health concern.	17
Public health emergency	•Evaluate decision-making to allocate resources during a public health emergency. •Critique public's perception of a common good during an emergency. •Integrate equity in public health emergency resource allocation.	13
Global perspective	•Apply strategies in global health finance. •Critique universal health coverage financing strategies. •Examine universal financial protection policies in LMICs.	13

## Results

While a robust assessment and presentation of evaluation data is beyond the scope of this paper, students were provided the space and opportunity to provide feedback on the class content and materials. This allowed for valuable input that we intend to incorporate as we update and improve both course content and delivery. Feedback received to date included:

- Guest lecturers with both subject matter expertise and experience in public health finance should be engaged. Because public health finance is such a broad topic area, it is difficult for any one person to bring expertise and experiences relevant to the range of topics and public health settings. Including outside experts to provide guest lectures and/or serve as “the expert in the room” to facilitate content-related discussions provides an opportunity for students to learn from a range of experts with diverse experiences and knowledge.
- A stronger emphasis on management practices and tools should be provided. The current curriculum has a substantial amount of content on budgeting and accounting practices. Exposure to management approaches and examples on how to manage and administer funds can be a valuable contribution to the course.
- Providing additional examples from state and local context as well as illustrations from the national or federal level cultivates a more well-rounded course. An emphasis should also be given to providing examples and case studies on U.S. tribes and territories, given the unique contexts in which tribes and territories operate.
- Leveraging the expertise in the room should be highlighted. As adult learners and practicing professionals, DrPH students may have applicable experiences as well as examples that help highlight the relevance of the course content. Providing an open space for discussion, dialogue, and constructive debate between students helps to reinforce the content.
- Given the political nature of the funding process it is important to include news articles to stay abreast of current events and the political dialogue. This also consists of various modes of materials like news articles, podcasts, webinars to suit various types of learning styles.

## Discussion

Many public health agencies do not carry out systematic financial analysis as part of their planning or review process. This threatens public health agencies' ability to quantify their fiscal condition and the sustainability and state of readiness of the public health system when an outbreak or natural disaster occurs (12). An analytical tool to measure financial performance in local health departments was recently introduced to help mainstream and apply analytical concepts to the practice of public health management (12). While measuring financial performance is not a requirement, the benefits have implications for policy and practice, including the creation of a uniform chart of accounts to help assess the financial condition of the public health system. This enables public health managers and leaders to forecast national trends, make informed decisions for the continuation or elimination of programs, increase financial accountability, and advocate for additional funds (12).

Until public health agencies prioritize financial performance, the field will continue to experience what the World Bank penned as the “cycle of panic and neglect” (13). This term describes the reactionary response from political leaders to invest in public health systems during a time of need until the urgency fades (13). Public health professionals work and adapt in this unpredictable environment. System change will require preparing future public health leaders about these inherent challenges and innovative ways to efficiently manage funds and advocate for sustainable public health funding.

Based on our review, current academic content for doctoral-level students does not meet the practical applications public health professionals are expected to provide in the workplace. The current academic content in public health finance falls short of providing the necessary skills and strategies to effectively mobilize and manage funds in public health. Courses have content that is disproportionately health care-focused and fails to meaningfully address finance policies and management strategies unique to public health. Most current curricula are inadequate and did not properly prepare DrPH students to assume leadership and management functions, including identifying sources of public and private funds and aligning programming and allocation to meet the organization's needs and mission.

Honoré and colleagues outlined potential impediments to developing a management and finance course for public health. Those included the limited number of credit hours available in the curricula, a lack of dedicated faculty with the academic credentials, experience, and time to develop a specific public health finance course, and the lack of teaching materials dedicated to public health finance (2). When dedicated faculty are not feasible, they suggest using adjunct faculty to conserve full-time faculty resources. For DrPH students, in particular, having faculty engaged in applied practice, provides a valuable opportunity to include real-world and real-time case studies. This paper and corresponding appendix help to alleviate many of the barriers to course development and relevant teaching materials.

Based on evaluation feedback from the initial piloting of this course in Georgia State University's School of Public Health during the 2021–2022 academic year showed early signs of success in achieving course objectives. We recognize that continuous improvements to course curriculum are not only valuable but needed to keep up with the latest current events. We plan to incorporate feedback into the next iteration of the curriculum and plan to continue receiving informal and formal feedback through post-course evaluations.

Preparing and strengthening public health leadership in management and finance through a doctoral-level course will not alleviate the systemic challenges facing public health funding and infrastructure. However, aligning academic competencies and training with professional responsibilities is critical to positioning public health organizations and agencies for greater impact and improved population health outcomes. Preparing public health leaders to participate more effectively in budget, financing, and funding strategies and discussions also positions them to justify and advocate for additional public health resources with decision makers and appropriators.

## Constraints

The largest constraints to development and implementation of a public health-focused doctoral-level finance and management course were finding relevant course materials, including textbooks, articles, assignments, and curricular content, like learning objectives and lesson plans that were pertinent to applied public health practice. Another practical constraint was that the course was initially administered during the 2021–2022 academic year coinciding with the COVID-19 pandemic. As a result, classes were held in a synchronous remote learning environment. While this mode helps with engagement of guest lecturers outside the geographic area, it inherently limits the faculty's ability to encourage dialogue, debate, and discussion between students. Moving forward, we plan to administer the class using a hybrid approach with half the classes meeting in-person to facilitate open dialogue and discussion and the other half using a synchronous remote environment to facilitate engagement of outside guest lecturers. Lastly, a major constraint that other schools of public health may face in adapting this curriculum is finding dedicated faculty with the academic credentials, experience, and time to teach this course. As noted above, identifying adjunct faculty with the requisite background in public health finance is challenging, but ideal for optimal course delivery.

## Conclusion

Preparing the next generation of public health leaders, not only for the demands of current public health practice, but for the essential roles public health must play in collaborating to address upstream social determinants of health is a critical charge for DrPH training programs. Improving population health and advancing health equity require core competencies in both the cultivation and deployment of public health financial resources. This paper describes the development of a public health management and finance course designed to strengthen finance and management knowledge and skills for future public health leaders. The course aims to prepare doctoral-level students to recognize the inherent challenges of public health finance while also providing finance and management knowledge and skills essential for successful public health leadership and practice.

## Data availability statement

The original contributions presented in the study are included in the article/Supplementary material, further inquiries can be directed to the corresponding author.

## Author contributions

All authors listed have made a substantial, direct, and intellectual contribution to the work and approved it for publication.
