# Supplementary material for: Developing a management and finance training for future public health leaders
Source: Front Public Health. 2023 May 11;11:1125155. doi: 10.3389/fpubh.2023.1125155 (PMC10213770; doi:10.3389/fpubh.2023.1125155)
Supplement: Supplementary file 1 [file Table_1.DOCX]

**Appendix A. Public Health Finance & Management Curriculum**

| 1. Introduction |
| --- |
| Provides the basic foundation of public health finance including theories, concepts and definitions and an overview of funding strategies in the Public Health 3.0 paradigm.  CEPH Competency: #12: Propose human, fiscal and other resources to achieve a strategic goal  Learning Objectives:   - Summarize fundamental theories, concepts, and definitions of public health finance - Apply strategies for public health financing in Public Health 3.0 - Self-evaluate personal experience and expectation to improve knowledge and skills in finance & management   Proposed Articles  Honore, P., Amy, B. (2007)  J Public Health Manag. Pract. Vol 13, Iss 2 p 89-92    Department of Health and Human Service (2016) A Call to Action to Create a 21^st^ Century Public Health Infrastructure.  Pages 20-21    Levi, J., DeSalvo, K. (2017) “Funding for Local Public Health: A Renewed Path for Critical Infrastructure” Health Affairs |
| 1. Planning and Budgeting |
| Describes the process of congressional appropriations for public health funds and types of funding mechanisms and strengthen skills in budget development.  CEPH Competency: #12: Propose human, fiscal and other resources to achieve a strategic goal  Learning objectives   - Discuss congressional appropriation process in theory and in practice - Analyze differences in U.S. state public health funding levels - Critique funding mechanisms and types of revenue source for federal, state, and local levels   Proposed Articles  Hawkins, D. (2017). How Appropriations are Supposed to Work. Roll Call    McCullough JM. (2019) “Decline in Spending Despite Positive Return on Investment: Understanding Public Health’s Wrong Pocket Problem” Frontiers of Public Health    Public Health Finance and Management. Public Health Finance Boot Camp Module I. |
| 1. Operating Budgets |
| Describes types of budgets and differences in cost types and provides real-world perspective on timing of funds and mechanisms to achieve a strategic goal.  CEPH Competency: #12: Propose human, fiscal and other resources to achieve a strategic goal  Learning Objectives   - Evaluate the overall planning process and implementing an operating budget - Design a budget using different types of costs and budget assumptions - Analyze a planning and operating budget   Articles  Public Health Finance and Management. Bootcamp Module II. Estimating Costs and Margins.    Module III Financial Planning and Budgeting    Khan Academy cash vs accrual basis |
| 1. Financial Statements |
| Demonstrates use of financial statements to evaluate programs, different types of financial statements, and distinction between assets and liabilities.  CEPH Competency: #12: Propose human, fiscal and other resources to achieve a strategic goal  Learning Objectives   - Calculate break-even analysis and discounted cash flow analysis - Evaluate financial statements and key players in financial statement regulation - Design a planning budget for a public health program   Articles  Public Health Finance and Management. Bootcamp Module IV Financial Evaluation of a New Program    Public Health Finance and Management. Bootcamp Module V Financial Reporting |
| 1. Taxation |
| Describes use of taxes subsidies to achieve public health goals, strategies to pass taxes, and regressive versus progressive tax structures.  CEPH Competency: #13: Cultivate new resources and revenue streams to achieve a strategic goal  Learning Objectives   - Evaluate taxation as a revenue source to support public health goals - Compare and contrast states with different tax structure and use of revenue - Design an operational budget for a public health program   Articles  Bownwell K., Farley, T., et al. (2009) “The Public Health and Economic Benefit of Taxing Sugar-Sweetened Beverages” The New England Journal of Medicine 361;16    Bird, B. (2019) “What is the Soda Tax and Which Cities Have One?” The Balance    Harker, L. “Increase the State Tobacco Tax for a Healthier Georgia” (2018) Georgia Budget & Policy Institute    Mozaffarian, D., Rogoff K., Ludwig, D. (2014) “The Real Cost of Food. Can Taxes and Subsidies Improve Public Health?” JAMA 312(9):889-890 |
| 1. Revenue Generation |
| Describes innovative models to generate funds and public health agencies’ ability to braid and blend funds to support a public health mission.  CEPH Competency: #13: Cultivate new resources and revenue streams to achieve a strategic goal  Learning Objectives   - Explain social impact bonds and develop a real-world example - Critically examine sustainability and utility of new economic models - Design a budget that blends and braid funds to address upstream social determinants of health   Articles  Eccles, Toby (2013). Investing in Social Change. TedTalk    Rabinowitz, D., Barrett, M., Snebold, L. (2016) “Overview of Innovative Funding Mechanisms for Public Health” Presented at the 12^th^ Public Health Finance Roundtable |
| 1. Spending and Return on Investments (ROI) |
| Provides an overview of public health spending, measuring return on investment, and communicating public health effectiveness.  CEPH Competency #17: Propose interprofessional team approaches to improving public health  Learning Objectives   - Discuss evidence-based resource allocation processes - Examine real world resource allocation processes - Apply equity in a resource allocation decision-making process   Articles  Honore, P., Fos, P., Smith, T., Riley, M., Kramarz, K. (2010) “Decision Science: A Scientific Approach to Enhance Public Health Budgeting”. J. Public Health Manag. Pract. Vol 16, Iss 2, pages 98-103.    Buehler, JW., Holtgrave, DR. “Who gets how much: Funding formulas in federal public health programs.” J. Public Health Manag. Pract.  Vol 12 Iss 2 Pages 151-155.  Joseph, KT., Rice, K., Li, Chunyu. (2016) “Integrating Equity in a Public Health Funding Strategy” JPHM&P. 22 Supp 1 S68-S76 |
| 1. Fiscal Stewardship & Transparency |
| Provides an overview of systems that support fiscal stewardship and reporting to ensure transparency.  CEPH Competency: #13: Cultivate new resources and revenue streams to achieve a strategic goal  Learning Objectives   - Examine public health expenditure data sources - Assess the relationship between resource allocation and health equity goals - Critique funding policy using the Tobacco Master Settlement Agreement as an example   Articles  Leider, JP., Resnick, B., Bishai, D., Scutchfield, D. (2018) “How Much Do We Spend? Creating Historical Estimates of Public Health Expenditures in the United State at the Federal, State and Local Levels” Annual Review of Public Health vol 39 p 471-87    McCullough, JM., Leider, JP. (2016) “Government Spending in Health and Non-health Sectors Associated with Improvement in County Health Rankings.” Health Affairs Vol 35, No. 11 |
| 1. Partnerships |
| Describes partnerships that blend and braid funds in support of public health initiatives and goals.  CEPH Competency: #13: Cultivate new resources and revenue streams to achieve a strategic goal  Learning Objectives   - Discuss fiscal stewardship, accountability and transparency of public health funds - Critique public health programming and closing the finance gap - Compare and contrast state-level expenditures in social services and social determinants of health   Articles  Honore, P., Zometa, C., Thomas, C., Edmiston, A. (2019) “The Public Health Uniform National Data System (PHUND$) A platform for monitoring fiscal health and sustainability of the public health system. J. Public Health Manag. Pract. Vol 25. Iss 4 pg    Honore, P., Clarke, R. et al. (2007) “Creating Financial Transparency in Public Health: Examining Best Practices of System Partners. J. Public Health Manag. Pract. 13(2):121-9 |
| 1. Decision-Making Strategies |
| Describes approaches to allocating funds and use of evidence to inform decision-making, including use of decision analysis software, performance-based funding, formula-based funding, and equity measures for resource allocation  CEPH Competency #17: Propose interprofessional team approaches to improving public health  Learning Objectives   - Analyze Medicaid’s role in partnering with public health - Evaluate the intersection between public health and safety net programs to improve social determinants of health - Assess public health agencies’ ability to blend funds to support people living with disabilities   Articles  Somers, S., Crawford, M. (2017) “Medicaid-Public Health Partnership: Untapped Potential to Improve Health Care and Reduce Costs.” Center for Health Care Strategies, Inc.    Beers, A., Moses, K. (2019) “A Marriage between Medicaid and Public Health: A Q&A on Partnering for Prevention” Center for Health Care Strategies. Inc.    Harker, L. (2018) State Earned Income Tax Credit. A Proven Tool to Improve Health |
| 1. Ethics |
| Describes the implications of advocating, receiving, and managing funding from philanthropy and private corporations.  CEPH Competency #17: Propose interprofessional team approaches to improving public health  Learning Objectives   - Examine the influence of philanthropic and private industry funds in public health - Evaluate the intersection of pharmaceutical research and public health research and practice - Critique the decision-making process in accepting funds to address a public health concern   Articles  Stuckler, D., Basu, S., McKee, M. (2011) “Global Health Philanthropy and Institutional Relationships: How Should Conflicts of Interest Be Addressed?” PLoS Medicine 8(4)    Berman, R. (2020) “Where are the Billionaires?” The Atlantic    Rabin R. (2018) Federal Agency Courted Alcohol Industry to Fund Study on Benefits of Moderate Drinking. New York Times. |
| 1. Public Health Emergency |
| Describes resource allocation during an outbreak, emergency, or humanitarian response, philosophical perspectives that determine the distribution of resources, and changes in the public’s perspective of a public good.  CEPH Competency: #13: Cultivate new resources and revenue streams to achieve a strategic goal  Learning Objectives   - Evaluate decision-making to allocate resources during a public health emergency - Critique public’s perception in a common good during an emergency - Integrate equity in public health emergency resource allocation   Articles  Strosberg, M. (2006) “Allocating scarce resources in a pandemic: Ethical and public policy dimensions.” AMA Journal of Ethics.    Pagel, C., Utley, M., Ray, S. (2020) “COVID-19: How to triage effectively in a pandemic” the bmj opinion.  Frakt, A., (2020) “Who Should be saved first? Experts offer ethical guidance” New York Times.    Emanuel, E., Persad, G., et al. (2020) “Fair allocation of scarce medical resources in the time of COVID-19.” The New England Journal of Medicine. |
| 1. Global Perspective |
| Provides resource allocation funding strategies in a global context with a particular emphasis on universal health coverage  CEPH Competency: #13: Cultivate new resources and revenue streams to achieve a strategic goal  Learning Objectives   - Apply strategies in global health finance - Critique universal health coverage financing strategies - Examine universal financial protection policies in LMICs   Articles  Sen, A. (1999). “Health in Development.” Bulletin of the World Health Organization, vol. 77, no. 8, pp. 619-623.    Kelly, R., Hemming, R., Bharali, I., Glenday, G., Asfaw, A. (2020) “Public Financial Management Perspective on Health Sector Financing and Resource Allocation in Ethiopia.” Duke Global Working Paper Series No. 18.  Ghebreyesus, TA. (2019) Future of global health financing: Hope vs. Reality in the push for universal health coverage.  Soucat, A. (2020). Global Common Goods for Health: Towards a New Framework for Global Financing. Global Policy |
